# Safety of Continuous Infusion Ketorolac in Postoperative Coronary Artery Bypass Graft Surgery Patients

**DOI:** 10.3390/pharmacy4030022

**Published:** 2016-06-28

**Authors:** Meredith L. Howard, Robert D. Warhurst, Courtney Sheehan

**Affiliations:** 1 University of North Texas System College of Pharmacy, 3500 Camp Bowie Blvd, Fort Worth, TX 76107, USA; 2 Department of Pharmacy, Indiana University Health, Saxony Hospital, 13000 E. 136th St., Fishers, IN 46037, USA; Rwarhurst@iuhealth.org; 3 Department of Pharmacy, Indiana University Health, Methodist Hospital, 1701 N. Senate Ave., AG401, Indianapolis, IN 46202, USA; Ckhouli@iuhealth.org

**Keywords:** coronary artery bypass graft, surgery, ketorolac, continuous infusion, pain, safety

## Abstract

Background:Continuous infusion ketorolac is sometimes utilized for analgesia in postoperative coronary artery bypass graft (CABG) patients despite contraindications for use. Limited literature surrounds this topic; therefore, this study was conducted to evaluate the safety of this practice. Methods: This retrospective cohort study evaluated the primary outcome of mortality and secondary outcomes of incidence of bleeding and myocardial infarction (MI). All patients who underwent isolated CABG surgeries and received continuous infusion ketorolac during the study period were included. An equal number of randomly selected isolated CABG patients served as control patients. Electronic medical records and the Society of Thoracic Surgeons (STS) database were utilized to determine baseline characteristics and outcomes; Results: One hundred and seventy-eight patients met inclusion; 89 in each group. More patients in the control group underwent on-pump surgeries (78.6% vs. 29.2%, *p* = 0.01) and had higher STS risk scores (1.1% vs. 0.6%, *p* = 0.003). There was no difference in mortality between the ketorolac group and control group (2.2% vs. 3.3%, *p* = 0.605). Additionally, no patients experienced a MI and there was no difference in bleeding incidence (5.5% vs. 6.7%, *p* = 0.58); Conclusions: No association was found between continuous infusion ketorolac and increased risk of mortality, MI, or bleeding events in postoperative CABG patients. Considerations to differences in baseline characteristics must be made when interpreting results.

## 1. Introduction

Non-steroidal anti-inflammatory drugs (NSAIDs), including ketorolac, contain a boxed warning for increased risk of cardiovascular thrombotic events, myocardial infarction (MI), and stroke. Additionally, ketorolac is contraindicated for pain control in the setting of coronary artery bypass graft (CABG) surgery because of the risk for increased bleeding and cardiovascular adverse events [1]. Despite these risks and warnings, ketorolac is frequently used as an additional analgesic modality in postoperative CABG patients. 

A multi-center, randomized, double-blind, placebo controlled study of the oral cyclooxygenase-2 (COX-2) selective NSAID, valdecoxib, and its intravenous (IV) prodrug, parecoxib, was published in 2005 to evaluate safety endpoints in 1671 postoperative CABG patients. Known risks of gastric ulceration, renal injury, and bleeding, as well as previously reported serious adverse events associated with the use of these drugs provoked this investigation. A statistically significant increased risk of cardiovascular events was found in postoperative CABG patients with use of the COX-2 selective NSAID compared with placebo [2]. Cardiovascular events included MI, sudden cardiac death, ischemic or hemorrhagic cerebrovascular accident, deep vein thrombosis, and pulmonary embolism. These findings and lack of long-term safety data in all NSAIDs prompted withdrawal of valdecoxib from the market as well as the Food and Drug Administration boxed warning for cardiovascular adverse events on all COX-2 selective and non-selective NSAIDs [3]. This also resulted in a contraindication for the use of NSAIDs in the setting of CABG surgery.

The boxed warning and contraindication associated with ketorolac and other NSAIDs has not precluded their use, including in cardiothoracic surgery patients. Typically, ketorolac is administered intermittently, but may also be administered off-label as a continuous infusion, a practice that is conducted at our institution in postoperative CABG patients. Continuous infusion ketorolac has already been shown to decrease opioid use and opioid associated adverse effects in other patient populations; however, the concern still exists for risk of cardiovascular adverse effects [4,5,6]. Intermittently dosed ketorolac has been evaluated in CABG and cardiovascular surgery patients and has not shown untoward adverse cardiovascular events [7,8,9]. In addition to safety, various cardiovascular and mortality benefits have been seen with intermittent ketorolac in previous studies, both in cardiovascular and non-cardiovascular patient populations [7,8,10,11].

Although several studies have shown no risk and possible benefits of intermittent ketorolac in various surgical settings, there is a need to evaluate certain risks that may be associated with continuous infusion use in CABG patients, a population that is at a high risk of cardiovascular related adverse events. No studies evaluating continuous infusion ketorolac in this setting have been identified; therefore, it is necessary to determine if findings from intermittent studies can be applied to continuous infusion. The purpose of this study is to assess the influence of continuous infusion ketorolac on mortality and to assess its safety with respect to bleeding risk and myocardial infarction in postoperative CABG patients.

## 2. Materials and Methods

This study was a single-center, five-year, retrospective chart review of patients who underwent isolated CABG surgeries between 1 January 2008 and 31 December 2012, as far back as data could reliably be retrieved from the electronic medical record (EMR). All patients who received continuous infusion IV ketorolac following an isolated CABG surgery were included in the treatment arm of the study. Patients were identified via EMR reports and ketorolac administration was verified with the electronic medication administration record (EMAR). An equal number of isolated CABG patients who did not receive ketorolac were selected by random number generator for inclusion in the control group. Patients who underwent any surgery in addition to CABG were excluded to eliminate any confounding factors related to additional surgery type. Additionally, the protected populations of prisoners, pregnant patients, and patients under the age of 18 were also excluded. This study was approved by the Institutional Review Board.

Study patients received ketorolac in accordance with our institution’s order set. Per the thoracic postoperative order set, ketorolac continuous infusion is a selectable option for pain management. It is started postoperatively and is administered at a rate of 3.6 mg per hour from an admixture of 90 mg of ketorolac in 1000 mL of normal saline. A bolus may be given at the start of therapy if desired. The infusion is continued for a total of 24 hours (90 mg ketorolac maximum). 

Electronic medical records and the institution’s Society of Thoracic Surgeons (STS) database were utilized to collect demographic information as well as pertinent surgical data such as operative pump type and STS risk score on all patients. The primary outcome evaluated in this study was mortality which included both hospital mortality and 30-day all-cause mortality. Secondary outcomes included postoperative myocardial infarction as documented by a physician in the EMR, change in hemoglobin and platelet count from preoperative baseline, and clinically significant bleeding as noted in the patient’s EMR. The Thrombolysis in Myocardial Infarction (TIMI) criteria were used to define clinically significant bleeding, which includes chest tube output greater than two liters in 24 hours, transfusion of greater than or equal to five units of whole blood or packed red blood cells within a 48-hour period, perioperative intracranial bleeding, surgical re-exploration for the purpose of controlling bleeding, or fatal bleeding [12]. 

Statistical analysis was completed using SPSS version 21 (Chicago, IL, USA). The chi-squared test or Fisher’s exact test were used for dichotomous variables including hospital mortality, 30-day all-cause mortality, MI, and clinically significant bleeding. Continuous data was analyzed using the independent t-test or Mann-Whitney U. No power calculations were made as this was a retrospective study that included all-comers for the active arm. 

## 3. Results

**Figure 1:**
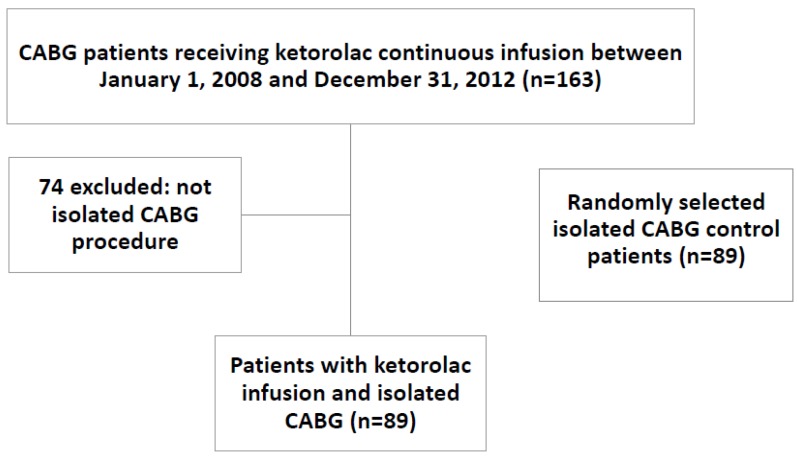
Patient Enrollment and Eligibility.

One-hundred and sixty-three patients underwent CABG surgery and received continuous infusion ketorolac. Seventy-four of these patients were excluded as they did not undergo an isolated CABG surgery. Ultimately, 89 patients who underwent an isolated CABG surgery and received continuous infusion ketorolac were included in the study. An additional 89 isolated CABG patients who did not receive ketorolac were included in the control arm for a total of 178 patients. Details of patient inclusion can be seen in Figure 1 and baseline characteristics of the two groups is shown in Table 1. Characteristics were similar between groups with respect to age, gender, smoking status, and outpatient use of antiplatelet agents or NSAIDs. There was a statistically significant difference between patients who underwent an on-pump CABG surgery: 29.2% in the ketorolac group and 78.6% in the control arm (*p* = 0.01). Additionally, STS risks scores were significantly different between the groups: 0.6 (0.4–1.2) (median %, IQR) with ketorolac versus 1.1 (0.4–2.4) for the control arm (*p* = 0.003).

No significant difference in the primary outcome of mortality was found between the two groups. Death occurred in 2.2% of patients in the ketorolac arm and 3.3% of patients in the control arm (*p* = 0.605) for both hospital and 30-day mortality. No patients in either group experienced a postoperative MI. There was no difference between groups with respect to clinically significant bleeding and change in hemoglobin and platelets. Primary and secondary outcomes results can be found in Table 2.

## 4. Discussion

Continuous infusion ketorolac, often used in postoperative surgical patients as an additional modality for pain control, has limited literature surrounding its use in cardiothoracic surgery. Use of this agent, despite a boxed warning which includes increased risk of cardiovascular thrombotic events and potent antiplatelet properties, has spurred debate and interest surrounding its use. Because of the associated warnings, the outcomes investigated in this study included risk of death, MI, and bleeding events. The results of this retrospective study showed no differences in the safety outcomes of mortality, bleeding risk, and post-operative MI between continuous infusion ketorolac use and control patients. 

Similar safety outcomes have been evaluated in both CABG and non-cardiothoracic surgery populations. In CABG patients, two retrospective studies describe intermittent IV ketorolac safety outcomes. One study described an association between ketorolac use and lower 90-day mortality compared with no ketorolac use [7]. The second study evaluated graft closure on follow-up coronary angiography and showed lower rates of graft closure as well as a lower risk of mortality, though not a primary endpoint, with ketorolac compared with no ketorolac use [8]. Patients received ketorolac 15 to 30 mg every 6 hours per surgeons’ discretion in both studies. A third, larger, single-center retrospective study evaluated the ketorolac boxed warning in postoperative cardiac surgery patients (including CABG, valve surgery, and atrial fibrillation Maze procedures). Ketorolac was dosed at 15 to 30 mg every 6 hours as needed and the mean dose administered was 81.7 ± 52.3 mg. After adjustment in a multivariate model, intermittent ketorolac was not associated with an increase in the composite outcome of perioperative stroke, transient ischemic attack, MI, renal insufficiency, gastrointestinal bleeding, or death (odds ratio 0.72; *p* = 0.23) [9].

Beneficial opioid sparing effects of continuous infusion ketorolac have been documented outside of the cardiothoracic surgery population [4,5,6]. Cardiovascular outcomes associated with intermittent and continuous infusion ketorolac have also been evaluated in general medical and orthopedic patients. With respect to MI risk in general hospitalized patients, intermittent IV ketorolac was associated with a reduced risk of developing an MI while receiving ketorolac and for up to 3 days after therapy [10]. Of a similar effect, continuous infusion ketorolac use was associated with a decreased duration but not incidence of myocardial ischemic attacks post-surgery in postoperative elective total hip or knee arthroplasty patients [11].

**Figure 2:**
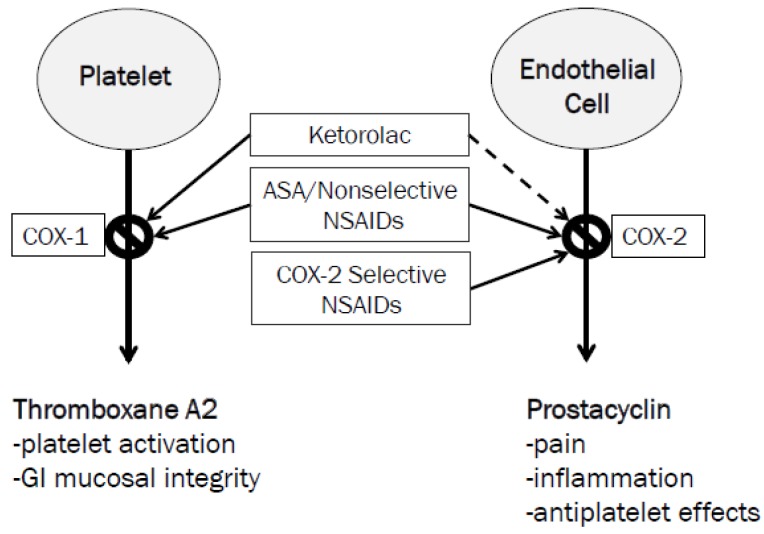
Cyclooxygenase (COX)-1 and COX-2 Activity and non-steroidal anti-inflammatory drugs (NSAID) Selectivity.

The hypothesis by which ketorolac exerts these possible beneficial effects is proposed to be related to its COX-1 selectivity and minimal inhibition of COX-2 [13]. As previously discussed, the boxed warning for NSAIDs arose from specific data for the COX-2 selective NSAID, valecoxib [2,3]. COX-2 inhibitors selectively reduce prostacyclin synthesis with no effect on thromboxane A2 (Figure 2). Prostacyclin is a potent inhibitor of platelet aggregation; its selective blockade by COX-2 inhibitors may upset thrombosis homeostasis and cause adverse cardiovascular events. Ketorolac, on the other hand, potently blocks platelet aggregation through thromboxane A2 inhibition [13,14]. This may be beneficial in patients with aspirin resistance to prevent CABG graft failure. The duration of this antiplatelet effect can last up to 24 hours after a single dose. Additionally, antiplatelet effects of ketorolac may outweigh the risk of bleeding in postoperative patients who may be hypercoagulable following specifically off-pump CABG surgery [8].

In addition to thromboembolic considerations, other risks with the use of NSAIDs must be noted in cardiovascular surgery populations. Other cardiac related adverse effects such as fluid retention and hypertension are also important to weigh when selecting to use an NSAID. The American Heart Association recommends a stepped-care approach to musculoskeletal pain management in patients with cardiovascular disease which can be generalized to other avenues of pain management [15]. Acetaminophen, aspirin, tramadol, and short-term opioid analgesics are recommended as first line agents. Use of non-selective NSAIDs or NSAIDs with increasing COX-2 selectivity should be considered only after failing first line agents, and with special considerations. The lowest possible dose of NSAID, and shorter durations should be used, ideally only in patients with low risk of thromboembolic events [15]. As in our patient population, although the ketorolac dosing was moderate at 90 mg total, it was limited to a duration of 24 h, an important consideration. 

Despite the data available from prior studies, no studies have been identified which evaluate safety outcomes associated with the use of continuous infusion ketorolac. Although the same drug is being administered, various considerations must be kept in mind when giving ketorolac by continuous infusion versus intermittently. Ketorolac labeling is only relevant for intermittent dosing. Additionally, pharmacokinetic parameters of continuous infusion ketorolac have not been identified and information is not known regarding possible drug accumulation and its effects on safety outcomes. Given these considerations, safety outcomes for intermittent dosing should not be generalized to continuous infusion.

Consistent with intermittent ketorolac literature, this study found no association between use of continuous infusion ketorolac and mortality, MI, or clinically significant bleeding. These findings, however, are limited by unforeseen differences in the baseline characteristics of number of on-pump CABG patients and STS risk scores. On-pump CABG refers to a patient placed on cardiopulmonary bypass during surgery whereas off-pump surgery is a more recent technique with grafting is performed as the heart continues to pump its own blood [16]. The STS risk score is calculated for all patients who undergo CABG surgery and serves as a predictor of post-operative mortality. The score takes into account a number of demographics and clinical variables including preoperative status, hemodynamics, type of surgery, and presence of previous cardiovascular interventions [17]. Scores are reported as a percentage risk conferring mortality and higher scores indicate a higher risk of mortality. Both pump type and STS risk score are important to consider when interpreting study results as they give a general indication of a patient’s severity of illness or risk of adverse outcomes following surgery. 

When considering differences in pump type and STS risk score, it is difficult to explain the influence that they may have on this study’s results. More patients with on-pump surgeries in the control arm may be due to selection bias leading to higher risk patients not receiving ketorolac. Generally, patients who receive on-pump CABG surgeries undergo more complex bypasses. Off-pump surgeries are typically reserved for patients who may have a less diseased heart, although the patients themselves may have more comorbidities. Higher STS scores in the control arm versus the ketorolac arm may also indicate selection bias because of the retrospective nature of this study. Control patients had a higher predicted risk of death; however, the patients who received ketorolac and control patients had similar rates of mortality. Although mortality was low in both groups, one possible hypothesis could be that ketorolac increases a patient’s risk of mortality to a point that it would be comparable to having an increased STS risk score. Thus, use of ketorolac versus an increased STS risk score would have comparable mortality rates as seen in this study. Further studies with more robust patient numbers are necessary to determine if no difference exists for certain. 

In addition to limitations in baseline characteristic differences, this study is limited by its retrospective nature and small sample size. As previously discussed, potential for selection bias cannot be ruled out. Randomizing the control patients was not enough to overcome this bias and future research should focus on equally matched patient characteristics, including pump type and STS risk scores, between groups. With respect to sample size, the small number of patients that received continuous infusion ketorolac over the study’s time span is difficult to overcome as all comers were included for the study arm. 

Finally, low incidence of outcomes resulted in a probable lack of power. Very few patients actually experienced the primary or secondary outcomes in this study. Although no differences were seen in any of the outcomes, there was likely not adequate power to show a difference. Considering the deficiency of literature for continuous infusion ketorolac in the CABG population, it was difficult to estimate the effect size of the outcomes. As a novel study, valuable information was gained regarding continuous infusion ketorolac in a previously unexamined patient population.

## 5. Conclusions

No association was identified between the use of continuous infusion ketorolac and mortality, MI, or clinically significant bleeding in postoperative CABG patients. Further research with equally matched groups and a larger number of patients is warranted to adequately determine the cardiovascular risks associated with continuous infusion ketorolac. 

## Figures and Tables

**Table 1:** Baseline Characteristics.

Characteristic	Ketorolac (n = 89)	Control Arm (n = 89)	*p*-Value
Age, mean ± SD	59 ± 8	62 ± 10	0.084
Male, n (%)	64 (71.9)	58 (65.1)	0.33
Smoker, n (%)	27 (30.3)	32 (35.9)	0.426
On pump CABG, n (%)	26 (29.2)	70 (78.6)	0.01
Outpatient use of NSAIDs or antiplatelets, n (%)	52 (68.4)	63 (77.7)	0.212
STS risk score, median % (IQR)	0.6 (0.4–1.2)	1.1 (0.4–2.4)	0.003

SD: standard deviation, n: number, CABG: coronary artery bypass graft, NSAIDs: non-steroidal anti-inflammatory drugs, STS: society of thoracic surgeons, IQR: interquartile range.

**Table 2:** Primary and Secondary Outcomes.

Outcome	Ketorolac (n = 89)	Control Arm (n = 89)	*p*-Value
Hospital mortality, n (%)	2 (2.2)	3 (3.3)	0.605
All cause 30-day mortality, n (%)	2 (2.2)	3 (3.3)	0.605
MI, n (%)	0 (0)	0 (0)	--
Clinically significant bleeding, n (%)	5 (5.6)	6 (6.7)	0.58
Decrease in hemoglobin, mean ± SD	4.6 ± 1.5	4.6 ± 1.8	0.61
Decrease in platelets, median (IQR)	74 (11–121)	72 (19–153)	0.17

n: number, MI: myocardial infarction, SD: standard deviation, IQR: interquartile range.
